# Caspase-3 Cleaves Extracellular Vesicle Proteins During Auditory Brainstem Development

**DOI:** 10.3389/fncel.2020.573345

**Published:** 2020-11-12

**Authors:** Forrest Weghorst, Yeva Mirzakhanyan, Kian Samimi, Mehron Dhillon, Melanie Barzik, Lisa L. Cunningham, Paul D. Gershon, Karina S. Cramer

**Affiliations:** ^1^Department of Neurobiology and Behavior, University of California, Irvine, Irvine, CA, United States; ^2^Department of Molecular Biology and Biochemistry, University of California, Irvine, Irvine, CA, United States; ^3^Section on Sensory Cell Biology, NIDCD, NIH, Bethesda, MD, United States

**Keywords:** auditory brainstem, neural development, caspase-3, non-apoptotic, extracellular vesicles, proteomics

## Abstract

Sound localization requires extremely precise development of auditory brainstem circuits, the molecular mechanisms of which are largely unknown. We previously demonstrated a novel requirement for non-apoptotic activity of the protease caspase-3 in chick auditory brainstem development. Here, we used mass spectrometry to identify proteolytic substrates of caspase-3 during chick auditory brainstem development. These auditory brainstem caspase-3 substrates were enriched for proteins previously shown to be cleaved by caspase-3, especially in non-apoptotic contexts. Functional annotation analysis revealed that our caspase-3 substrates were also enriched for proteins associated with several protein categories, including proteins found in extracellular vesicles (EVs), membrane-bound nanoparticles that function in intercellular communication. The proteome of EVs isolated from the auditory brainstem was highly enriched for our caspase-3 substrates. Additionally, we identified two caspase-3 substrates with known functions in axon guidance, namely Neural Cell Adhesion Molecule (NCAM) and Neuronal-glial Cell Adhesion Molecule (Ng-CAM), that were found in auditory brainstem EVs and expressed in the auditory pathway alongside cleaved caspase-3. Taken together, these data suggest a novel developmental mechanism whereby caspase-3 influences auditory brainstem circuit formation through the proteolytic cleavage of extracellular vesicle (EV) proteins.

## Introduction

The auditory system depends on specialized neural circuits that faithfully preserve sound information from the ears to higher-order auditory structures, processing various aspects of the sound environment along the way. Some auditory features, such as sound frequency, are neurally encoded as soon as the sound reaches the cochlea and are thereafter represented topographically throughout the auditory system. Other features, such as the spatial location of sound sources, are instead rapidly calculated by circuits in the auditory hindbrain and midbrain (Nelken, 2008). One such circuit in the auditory brainstem exploits interaural time differences (ITDs), discrepancies in the arrival time of sounds between the ears, to localize sound sources in horizontal space (Carr and Konishi, 1990; Overholt et al., 1992). Many species use this circuit to distinguish sounds arising from sources as close as one spatial degree, a feat that requires accurate detection of ITDs of less than 10 microseconds. Such extreme functional precision leaves little room for error in auditory neuroanatomy, which suggests that the auditory system employs high-fidelity systems of axon guidance and synapse formation to ensure that auditory projections innervate the correct targets. However, the molecular mechanisms responsible for assembling these precise circuits remain largely unknown.

We previously reported a requirement for the activity of the apoptotic protease caspase-3 for proper chick ITD circuit assembly (Rotschafer et al., 2016). This role was first suggested by the sequential, ascending expression pattern of active caspase-3 in neuronal processes of the chick auditory brainstem throughout embryonic development: First in axons of the auditory nerve (AN) on embryonic days (E) 6–7; then in axons of the AN synaptic target, *nucleus magnocellularis* (NM) on E8–10; and finally in dendrites of the NM synaptic target, *nucleus laminaris* (NL) on E11–13. When the caspase-3 activity was inhibited from E6 to E9, an examination of E10 brainstems revealed the mistargeting of NM axons and disruption of the laminar structure of NL. Importantly, TUNEL staining revealed no apoptotic cell death until after this time point, following earlier studies showing that the period of developmental cell death in the auditory brainstem begins around E11 (Rubel et al., 1976). Collectively, these findings suggest that caspase-3 influences chick ITD circuit development without causing cell death.

During apoptosis, pro-death signals result in the initiator caspases (i.e., caspase-2, -8, -9, and -10) activating the executioner caspases (i.e., caspase-3, -6, and -7) by cleavage of an inactive zymogen, or procaspase, form (Hengartner, 2000). The executioners then cleave a repertoire of protein substrates, culminating in cell death (Julien and Wells, 2017). Although caspases are typically associated with this role in apoptosis, several non-lethal functions of caspase-3 have been identified in the nervous system. Neurons become increasingly resistant to programmed cell death as they mature (Kole et al., 2013), and robust cellular safeguards allow caspase-3 to carry out other functions without leading to cell death (Hollville and Deshmukh, 2018). However, despite numerous examples of the importance of caspase activity in neural development and physiology (Campbell and Holt, 2003; Gulyaeva, 2003; Campbell and Okamoto, 2013; Ertürk et al., 2014; Wang and Luo, 2014; Unsain and Barker, 2015; Nakajima and Kuranaga, 2017), our understanding of non-apoptotic caspase function lags behind our knowledge of apoptotic caspase functions.

Because of the ease with which apoptosis can be induced in cell culture, hundreds of apoptotic caspase substrates have been experimentally verified (Lüthi and Martin, 2007; Crawford et al., 2012, 2013). Non-apoptotic caspase functions, in contrast, are often more transient and difficult to verify conclusively. Some studies have explored caspase activity in cellular compartments relevant to neural development, such as synaptosomes (Victor et al., 2018), but only a handful of substrates have been demonstrated to be important for non-apoptotic neurodevelopmental functions in living systems (Kellermeyer et al., 2018). An unbiased investigation of non-apoptotic neurodevelopmental caspase substrates that approximates the depth and breadth of screens for apoptotic substrates has never been accomplished. We therefore aimed to address the following outstanding questions: What are the proteolytic substrates of non-apoptotic caspase-3 activity during axonal guidance in the chick auditory brainstem, and how does the proteolysis of these substrates facilitate proper development of the ITD circuit?

We screened for caspase-3 cleavage events within a shotgun proteome of the auditory brainstem and found that many of the substrates of caspase-3 are associated with extracellular vesicles (EVs), membrane-bound nanoparticles that have important functions in intercellular communication. To verify this finding, we isolated EVs from the chick auditory brainstem and showed that they contain a large proportion of the proteins we identified as caspase-3 substrates. Finally, we found that two caspase-3 substrates associated with axon growth, Neural Cell Adhesion Molecule (NCAM), and Neuronal-glial Cell Adhesion Molecule (Ng-CAM), are sequentially expressed in axons and dendrites of the ascending auditory brainstem. This discovery parallels our previously published results on cleaved caspase-3 expression dynamics and potentially implicates non-apoptotic caspase-3 activity in the development of multiple structures in the chick auditory brainstem.

## Materials and Methods

### Chick Embryos

Chicken (*Gallus gallus domesticus)* eggs were obtained from a flock of White Leghorn roosters and Rhode Island Red hens (AA Laboratory Eggs). Eggs were incubated at 38°C on a tilting shelf cabinet incubator for 3 days before transfer to *ex ovo* cultures.

### *Ex ovo* Cultures

Eggs were cleaned with 70% ethanol, and their contents were transferred to a square weigh boat (Fisher). The weigh boat was covered with a plastic culture plate (Fisher) to prevent dehydration. *Ex ovo* cultures were returned to the incubator on a stationary shelf.

### Embryo Injection and Brainstem Dissection

After 6 days *in vitro* (DIV), embryos were staged at Hamburger-Hamilton stage 34 (corresponding to E9; Hamburger and Hamilton, 1951). The chorioallantoic and amniotic membranes were dissected and pulled aside, and the IV ventricle of each embryo was injected with a 50 μM solution of the caspase-3 inhibitor z-DEVD-fmk (R&D Systems, IC^50^ = 18 μM) or with vehicle solution (0.5% DMSO and 0.1% Fast Green in artificial cerebrospinal fluid). The injection was administered through a pulled glass capillary with a Picospritzer until the injection solution was seen in the tectum, about 5 μl. This injection procedure was repeated the following day (7 DIV; E10). Four hours after the second injection, the cultured embryos were harvested and brainstems were removed. The portion of the brainstem containing the auditory nuclei was dissected and snap-frozen at −80°C.

### EV Enrichment

EVs were isolated using a modified version of a previously described method (Vella et al., 2017). Briefly, embryos were incubated to E10, when auditory brainstems were dissected and snap-frozen as described above. Frozen brainstems were weighed in sterile microfuge tubes, then thawed and washed with Hibernate-E medium (Fisher) to eliminate any cell debris remaining from dissection or thawing. After the medium was discarded, the tissue was suspended in dissociation solution (7 μl/mg of tissue), consisting of Hibernate-E with 150 units/ml of collagenase-3 (Worthington Biochemical). Samples were placed in a 37°C water bath on a shaking platform for 5 min. Tubes were gently inverted several times to resuspend tissue then incubated in the shaking water bath for an additional 10 min. To release EVs embedded in the extracellular space of the brainstem tissue while avoiding cell damage or lysis, brainstem tissue was triturated gently by pipetting up and down several times with a sterile polypropylene P1000 filter tip (Corning). The dissociated tissue was returned to the shaking water bath for 5 min. To quench the collagenase digestion, PhosSTOP and Complete Protease Inhibitor Cocktail (Sigma) in Hibernate-E were added to the dissociate, for final concentrations of ~1X each. This quenching was followed by three serial centrifugation steps at 4°C (300× *g* for 5 min, 1,500× *g* for 10 min, and 10,000× *g* for 30 min). The supernatant was transferred to a new tube for each subsequent centrifugation. The supernatant from the 10,000× *g* step was pipetted into a 220-nm cellulose acetate centrifugal filter tube (Corning), which was spun for 30 min at 10,000× *g*. The filtrate was used for size exclusion chromatography.

EVs were enriched using a 35-nm Q26Voriginal column (IZON Science) according to the manufacturer’s instructions. The column was brought to room temperature then flushed with at least 15 ml of sterile, degassed PBS. The EV-containing filtrate (above) was pipetted onto the column. After the filtrate had entered the resin, sterile degassed PBS was added to begin elution. Once the void volume (3 ml) had eluted, six 0.5-ml fractions were collected, then four 1-ml fractions were collected, all according to the manufacturer’s instructions for a high-purity, low-yield EV sample. Protein in each fraction was quantitated using a Pierce modified Lowry assay (Thermo Fisher). The EV-containing fractions eluting 0–1.5 ml post-void were pooled and concentrated using an Amicon Ultra-2 ml centrifugal concentrator with 5 kDa MWCO (Millipore Sigma).

### NanoLC-MS/MS

Brainstem and EV samples were prepared as described above. Equivalent microgram amounts of these samples were incubated in 70% formic acid for 72 h at room temperature with constant shaking and occasional cup horn ultrasonication. One crystal of cyanogen bromide (CNBr) was added to each sample. After overnight incubation at room temperature in the dark, samples were evaporated to dryness in a Speedvac vacuum concentrator and re-dissolved in 8 M urea, 0.1 M triethylammonium bicarbonate (TEAB), and 10 mM tris (2-carboxyethyl) phosphine, pH 8.0, then incubated for 30 min at 37°C with occasional cup horn ultrasonication. Samples were then diluted to 6 M urea with 0.1 M TEAB, pH 8.0 followed by the addition of LysC at 1:100 (w/w) enzyme:protein ratio and incubation at 37°C. Samples were then diluted to 1 M urea with 0.1 M TEAB, pH 8.0 followed by the addition of Trypsin at 1:100 (w/w) enzyme:protein ratio. After overnight incubation at 37°C, samples were supplemented with formic acid to 2% final concentration. Peptides were desalted using C18/SCX as described (Rappsilber et al., 2007), eluting with 160, 205, 255, 325, 540, and 800 mM ammonium acetate in 20% acetonitrile, 0.5% formic acid, followed by a final elution with 5% NH_4_OH/80% CH_3_CN. Elutions were dried under vacuum and re-dissolved in 0.1% formic acid in water.

Using an Easy-nLC 1000 liquid chromatograph, a portion of each sample was injected to a 250 × 0.075 mm (ID) nanocapillary column packed in-house with ReproSil-Pur C18-AQ beads (1.9 μm diameter; Dr. Maisch GmbH). The column was eluted with a gradient of CH_3_CN in 0.1% formic acid (0–5% over 5 min extending to 25% over 205 min and to 35% CH_3_CN over a further 30 min) at a flow rate of 250 nl/min. The column eluate was sprayed into an LTQ Orbitrap Velos Pro mass spectrometer, collecting precursor spectra in the range 380–1600 m/z. Up to 15 of the most intense ions in each precursor spectrum with a charge of +2 to +4 and a minimum signal intensity of 5000 were fragmented by HCD with a normalized collision energy of 30%. Ions were dynamically excluded for 40 s after two fragmentations within 30 s *via* a 500-entry list, with early expiration from the list after detection within the exclusion period falling below S/N = 2.0.

Raw file data were processed to peak lists using Mascot Distiller 2.7.1. Using Mascot 2.6.1, each resulting mass list was subjected to target-decoy searching against the whole proteome of Gallus gallus (UniProt) plus a library of common contaminants, with semi-CNBr + Trypsin enzyme specificity, a maximum of two missed cleavages, parent and product mass tolerances of 20 ppm, and variable modifications of Oxidation (M), Deamidated (NQ), Carbamyl (N-term) and Met -> Hse (C-term M). Results were calculated using a threshold of *p* = 0.05 yielding an experimental false discovery rate (FDR) of < 3%. Matrices of peptides (rows) vs. samples (columns) were generated using in-house software.

### Identification of Caspase-3 Substrates

Mascot data were thresholded at an FDR of <0.03, and the resulting peptides were placed in a sample vs. accession matrix (Supplementary Data 1). The matrix was then filtered for peptides with a terminus of the form D↓X or E↓X (where D and E are aspartate and glutamate, the arrow represents the cleavage site, and X is any amino acid except proline) that were detected in at least one vehicle replicate but no z-DEVD-fmk (caspase-3-inhibited) replicates. D↓P was excluded because the sample was treated with formic acid, which has substantial D↓P specificity. Peptides meeting these criteria were considered to represent true caspase-3 cleavage sites.

### Sequence Logo

The sequence logo for auditory brainstem caspase-3 substrates (both D↓X and E↓X) was generated using IceLogo with the *Gallus gallus* proteome as a reference set and a cutoff alpha of 0.007, corresponding to an FDR of less than 1 amino acid (Maddelein et al., 2015).

### Functional Annotation Over-representation Analysis

Caspase-3 substrates were submitted for functional annotation term analysis with the Database for Annotation, Visualization, and Integrated Discovery (DAVID) Bioinformatics Resource 6.8 against a background of all proteins detected in the chick brainstem (Huang et al., 2009a,b). The default database settings on DAVID were used to identify enriched functional annotation terms, defined as any term with a Benjamini-adjusted *p*-value of less than 0.05 (Benjamini and Hochberg, 1995).

### ExoCarta Analysis

The relative probability of a protein appearing in an extracellular vesicle proteomics dataset was estimated using the total number of published EV datasets containing each protein on ExoCarta (Mathivanan and Simpson, 2009; Mathivanan et al., 2012; Simpson et al., 2012; Keerthikumar et al., 2016). Dataset counts included all proteins detected by mass spectrometry. Other protein evidence was excluded from this analysis to avoid artificial inflation of dataset counts for proteins commonly used as exosome markers on Western blots. Similarly, mass spectrometry datasets within the same study and with identical protein repertoires (due to a targeted proteomics approach) were counted only once. A Kruskal–Wallis test was used to compare EV dataset counts, and Dunn’s test was used for multiple comparisons.

### Protein Set Comparison

Official gene symbols of protein sets were submitted to the online tool Draw Venn Diagram (Bioinformatics and Evolutionary Genomics, Ghent University^1^) to obtain the numbers of unique proteins found in each set and their overlaps. This site was used to generate Figure 3 comparing ABC3 substrates, the brainstem proteome, and previously identified caspase-3 substrates (Lüthi and Martin, 2007; Rawlings et al., 2010; Crawford et al., 2013; Victor et al., 2018). For all other Venn diagrams, the numbers in set overlap from Draw Venn Diagram were used to create Venn diagrams with proportionally accurate areas using the Euler package on R.

**Figure 1:**
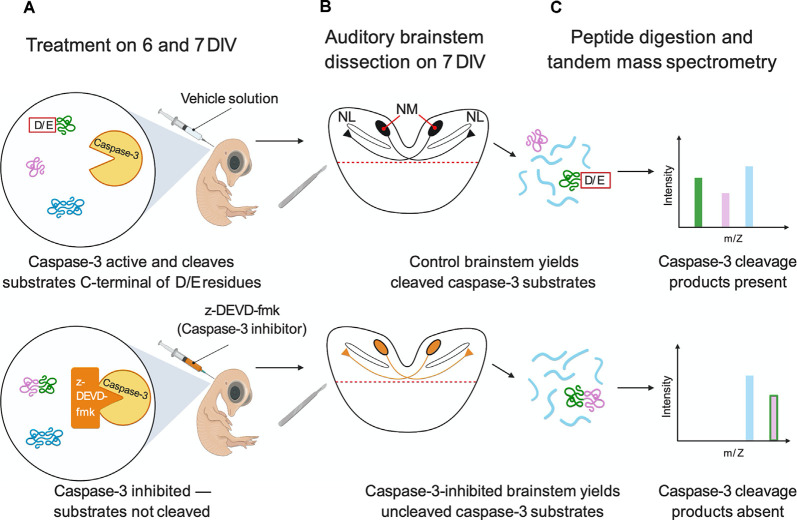
Experimental workflow for caspase-3 substrate identification. **(A)** Embryos were cultured *ex ovo* on E3 and were injected on 6 and 7 DIV with the caspase-3 inhibitor z-DEVD-fmk or vehicle solution. **(B)** Auditory brainstems (shown in coronal section) were isolated on 7 DIV. **(C)** Auditory brainstems (*n* = 3 replicates of two brainstems for each injection solution) were digested with trypsin and CNBr. Peptides were characterized by nanoLC-MS/MS. Likely caspase-3 substrates were identified as peptides with D/E↓X termini present in vehicle-injected replicates but not caspase-3-inhibited replicates. In the figure, the pink and green protein is a caspase-3 substrate because caspase-3 inhibition eliminates its caspase-3 cleavage products. The blue protein is not a caspase-3 substrate because it exhibits no change due to caspase-3 inhibition. Figure created with BioRender.com.

**Figure 2:**
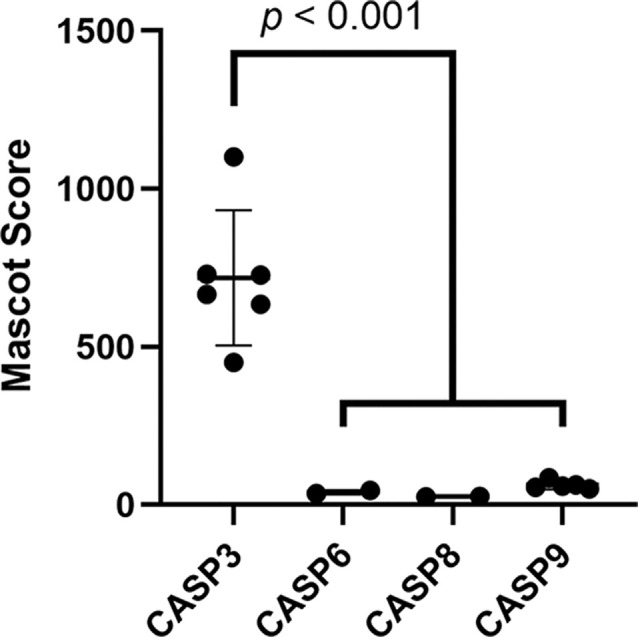
Caspase-3 is likely the most abundant caspase in the brainstem. Mascot scores of all caspases detected in the brainstem proteome were compared as a proxy for protein abundance. Caspase-3 had the highest Mascot score of all (Welch’s ANOVA with Dunnett’s multiple comparisons tests). Missing data points for caspase-6, -8, and -9 show that these proteins were not detected in all 6 brainstem proteomic replicates used to screen for ABC3 substrates.

**Figure 3:**
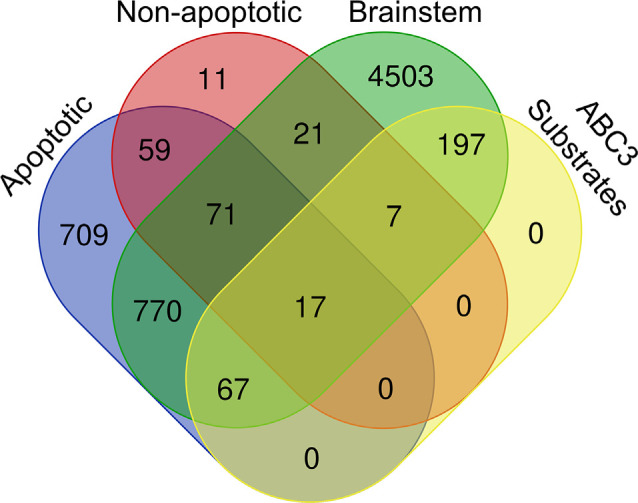
Venn diagram of the chick brainstem proteome, chick caspase-3 substrates, and known caspase-3 substrates, subdivided into Apoptotic substrates (MEROPS, CASBAH, and DegraBase Apoptotic) and Non-apoptotic substrates (DegraBase Untreated and Victor et al., 2018).

### Nanoparticle Tracking Analysis

EVs were purified from three biological replicates of 25–30 brainstems each, prepared on three different days as described above. Nanoparticle tracking analysis was performed as previously described (Breglio et al., 2020). Briefly, 150 μl (approximately 1/10^th^ of each sample) of each EV sample was diluted to 1.5 ml with PBS and filtered through a 0.2 μm PES syringe filter (Millex 33 mm, Sigma Millipore). A NanoSight NS300 controlled by NTA software version 3.1 (Malvern Panalytical) was used to measure the concentration and size distribution of EVs. Samples were pushed through a fluidic flow chamber at a constant flow rate using a syringe pump (Harvard Apparatus). The scattered light from vesicles illuminated with a 488 nm laser was recorded five times for 30 s at 30 frames/second using an sCMOS camera, keeping the camera sensitivity setting (13) identical between captures. Particle analysis was performed with a detection threshold of 3.

### Cryo-EM

EVs from approximately 30 brainstems were isolated as described above. Three μl of sample solution was applied to a glow-discharged Quantifoil grid (Quantifoil, R2/2) then loaded on a Leica EMGP plunger (Leica Biosystems). After blotting away excess liquid, the grid was quickly plunged into liquid propane. The cryo-grid was then transferred to a JEM-2100F electron microscope using a Gatan cryo-transfer holder (Gatan, Inc.). The electron microscope was operated at 200 KV with a field emission gun, and the sample was examined under minimum dose. The images were recorded with an OneView camera (Gatan, Inc.) at 50,000× magnification, corresponding to 2.16 Å/pixel resolution of specimen space. The “draw ellipse” function in ImageJ was used to measure vesicle diameters. The ellipse was fitted to the perimeter of each vesicle, whose diameter was taken as the average of the major and minor axes of the ellipse. A Mann-Whitney U-test was used to compare particle diameters from NTA and cryo-EM.

### Immunofluorescence

Eggs were incubated to E9 and E11 at 38°C in a tilting cabinet incubator. Embryos were removed and brainstems were dissected and fixed in 4% paraformaldehyde in PBS at 4°C for at least 1 h and up to overnight. Brainstems were incubated in 30% sucrose in PBS at 4°C overnight, then in a 1:1 mixture of (30% sucrose in PBS):(OCT Media) at 4°C until sectioning. Brainstems were then embedded in OCT media in a cryomold and were frozen at −20°C. Brainstems were sectioned coronally at the level of the auditory brainstem nuclei. Sections were cut at 12 μm and were flash-melted on subbed glass slides in a 1-in-4 series.

Slides with sectioned brainstems were rehydrated for 5 min in wash buffer (0.025% Triton X-100 in TBS), then solubilized for 10 min in 0.3% Tween-20 in TBS and washed with wash buffer for 3 × 10 min. Tissue was blocked for 1 h at room temperature in 10% normal goat serum and 1% BSA in TBS, then incubated overnight in primary antibody diluted in 1% BSA in TBS at 4°C (Table 1). Slides were immersed in wash buffer for 3 × 10 min, then incubated for 1 h in secondary antibody diluted in 1% BSA in TBS. Slides were washed in wash buffer for 3 × 10 min, then mounted with Glycergel (Dako-Agilent) and stored at 4°C until imaging. Multichannel fluorescent images were taken at 20× magnification with an Axiocam camera mounted on an Axioskop-2 epifluorescent microscope (Zeiss) using Axiovision software.

**Table 1:** Antibody concentrations.

Target	Source	Antibody Name	Immunofluorescence concentration	Western blot concentration
NCAM	DSHB	4d	3.5 μg/ml	0.35 μg/ml
NgCAM	DSHB	8D9	3.5 μg/ml	N/A
Cleaved caspase-3	Cell Signaling Technologies	9664	1:100	N/A
Caspase-3 (p12 subunit)	Abcam	ab179517	N/A	1:1,000
Calnexin	Abcam	ab13505	N/A	1:1,000
VDAC1	Abcam	ab154856	N/A	1:1,000
ApoA1	Invitrogen	PA5–21166	N/A	1:1,000

### Western Blotting

Three biological replicates of ABEV samples were prepared from 10–12 brainstems (30 mg of brainstem tissue) each as described above. The 300xg brainstem pellets were resuspended and dissolved by vortexing in RIPA buffer with Complete Protease Inhibitor and PhosSTOP (Roche). A Detergent-Compatible Bradford Assay was used to determine the protein concentration of crude brainstem lysates and ABEV samples. Brainstem lysates were diluted to concentrations approximately equal to those of the ABEV samples. Ten μg of protein from each sample were mixed with 4× Laemmli buffer (Bio-Rad) and 2-mercaptoethanol to a final concentration of 1X and 10%, respectively. Samples were loaded into an 8–20% Mini-PROTEAN TGX polyacrylamide gel (Bio-Rad). Together with a Chameleon Duo molecular weight ladder (LI-COR), samples were run in Tris/Glycine/SDS running buffer (Sigma Aldrich) for 1.5 h at 100 V and 20 mA. Gels were removed and equilibrated for 15 min in the Towbin buffer (Towbin et al., 1979). A PVDF membrane (Immobilon) was cut to size, activated in 100% methanol for 1 min, then equilibrated in Towbin buffer for at least 5 min. The gel and membrane were sandwiched between filter paper (Bio-Rad) and transferred in Towbin buffer at 100 V and 250 mA for 1 h. The membrane was removed, rinsed in nanopure water, and dried overnight. After reactivation in 100% methanol, the membrane was rinsed in nanopure water and incubated in REVERT Total Protein Stain for 5 min with gentle shaking. It was then washed twice for 30 s each in REVERT wash buffer (6.7% glacial acetic acid and 30% methanol in nanopure water) and imaged. Staining was reversed in REVERT reversal buffer (0.1 M NaOH and 30% methanol in nanopure water) for 15 min with gentle shaking. The membrane was rinsed briefly in nanopure water then blocked for 1 h at room temperature in Odyssey Intercept Blocking Buffer (Li-Cor). The membrane was incubated overnight at 4°C in primary antibody in Odyssey antibody dilution buffer with gentle rocking (Table 1). The membrane was washed three times for 5 min each in TBST (0.1% Tween-20 in Tris-buffered saline) then incubated for 1 hour at room temperature in Odyssey Intercept antibody diluent buffer with 0.01% SDS, goat anti-rabbit 800CW and goat anti-mouse 680RD secondary antibodies, each diluted 1:10,000. The membrane was washed three times for 5 min each in TBST, then rinsed in TBS and stored in TBS until imaging. The membrane was imaged wet at 169 μm resolution using ImageStudio on an Odyssey CLx blot scanner (Li-Cor). NewBlot IR Stripping buffer (Li-Cor) was used to strip the blot. The membrane was then rinsed two times for 5 min each in TBST, then rinsed three times for 5 min each in TBS before blocking and restaining in primary, as described above. Protein bands were normalized to total protein stain.

### Figure Design

BioRender was used to create Figure 1. Microsoft PowerPoint was used to draw proportionally accurate Venn diagrams, and “Draw Venn Diagram” was used to draw the 4-way Venn Diagrams in Figure 3 (Bioinformatics and Evolutionary Genomics, Ghent University^1^). Graphpad Prism 8 was used for all other graphs not otherwise mentioned.

## Results

### Identification of Caspase-3 Substrates in the Auditory Brainstem

To identify the targets of non-apoptotic caspase-3 activity in the embryonic chick auditory brainstem, we searched for likely caspase product peptides that were abolished by caspase-3 inhibition using the strategy shown in Figure 1. Specifically, we performed intraventricular injections of the caspase-3/7 inhibitor z-DEVD-fmk or vehicle solution at developmental stages when cleaved caspase-3 is expressed in NM axons and nowhere else in the auditory brainstem, namely embryonic days (E) 9 and 10, corresponding to Hamburger-Hamilton stages 35 and 36 (Hamburger and Hamilton, 1951; Rotschafer et al., 2016). We then used tandem mass spectrometry to characterize auditory brainstem proteins and peptides. We filtered the peptidomes for peptides likely produced by *in vivo* caspase-3 proteolysis based on two criteria:

(1) One peptide terminus was a canonical caspase cleavage site, with an aspartate (D) or glutamate (E) residue immediately N-terminal of the cleavage site (Seaman et al., 2016). Caspases represent 9 of the 10 chicken proteases with D/E↓X specificity, where ↓ represents the scissile peptide bond and X represents any amino acid. By contrast, CNBr and trypsin (which were used to digest the proteome before mass spectrometry) exhibit M↓X and R/K↓X and specificity, respectively.
(2) The peptide must have been identified in at least one of the vehicle replicates but in none of the three caspase-3-inhibited replicates, indicating that the caspase-cleaved terminus requires caspase-3 activity. This criterion was adopted based on the mass spectrometry “missing data” rubric, namely the enormously greater prevalence of incomplete proteomic data than of false-positive data from a sample under the conditions employed here (Karpievitch et al., 2012; Wei et al., 2018; McGurk et al., 2020). Thus, the detection of a peptide in some samples but not in others more likely reflects higher abundance (not false detection) of the peptide in the samples in which it was observed. Our set of substrates was denoted Auditory Brainstem Caspase-3 (ABC3) substrates.

We identified 421 distinct peptides that met both of the above criteria. This set included seven peptide pairs representing opposite sides of the same cleavage site, and three peptide pairs representing variable numbers of missed tryptic cleavages on the same side of the caspase cleavage site. Therefore, the 421 peptides were derived from 411 unique cleavage sites arising from 288 unique proteins, which represented about 5% of the total brainstem proteome of 5,653 unique proteins (Supplementary Data 1). Of the 288 likely ABC3 substrate proteins, 58 (20%) possessed multiple sites that fulfilled the two criteria for ABC3 cleavage, consistent with previous reports that many caspase substrates are cleaved at multiple sites (Crawford et al., 2013; Seaman et al., 2016).

### ABC3 Substrates Were Most Likely Cleaved by Caspase-3, Not Other Proteases

The inhibitor used in this study, z-DEVD-fmk, is designed to primarily inhibit caspase-3 and caspase-7 by its similarity to these caspases’ preferred consensus sequence. We detected caspase-3 but not caspase-7 in the chick auditory brainstem (Supplementary Data 1), suggesting that all peptides that fulfilled our criteria for ABC3 substrates are truly produced by caspase-3. However, several studies have shown that other caspases are inhibited by as little as 1 μM of z-DEVD-fmk (Berger et al., 2006; Timmer and Salvesen, 2007; Pereira and Song, 2008; Poręba et al., 2013; McStay, 2016), a concentration less than the inhibitor’s IC^50^ for caspase-3 (18 μM) and far below the 50 μM used in this study. Given this promiscuity of caspase inhibition, we considered whether other caspases detected in the auditory brainstem (caspase-6, -8 or -9) may have produced some of the cleavage sites that we attributed to caspase-3 activity. Several lines of evidence suggest that caspase-3 is responsible for the majority of ABC3 substrates. First, caspase-8 and -9 are initiator caspases with just 45 and 10 known substrates, respectively (Rawlings et al., 2010). Only one ABC3 substrate (vimentin) is also a substrate of caspase-8 or -9, so these initiator caspases are likely to be responsible for few, if any, ABC3 substrates. Additionally, though we did not conduct quantitative proteomic analysis, we used protein Mascot score, which correlates moderately with protein abundance (Ishihama et al., 2005), to compare the relative abundance of caspases in the auditory brainstem (Figure 2; Welch’s ANOVA, *p* = 0.0073). Caspase-3 had a greater Mascot score (mean = 717.8, 95% CI: 493.7–942.0) than caspase-6 (mean = 40.0, 95% CI: -23.5–103.5; Dunnett’s multiple comparisons test, *p* = 0.0006), caspase-8 (mean = 26.5, 95% CI: 20.2–32.9; *p* = 0.0005), and caspase-9 (mean = 62.6, 95% CI: 46.1–79.1; *p* = 0.0006), suggesting that caspase-3 is the most abundant caspase in the auditory brainstem. Indeed, the other three caspases were not observed in all six biological replicates used in this study, evidenced by missing Mascot score data. Taken together, these data suggest that inhibition of proteins other than caspase-3 did not contribute significantly to the identification of ABC3 substrates.

### Auditory Brainstem Caspase-3 Activity Cleaves Both Novel and Known Substrates, Especially Known Substrates Observed in Non-apoptotic Contexts

Next, we investigated whether our ABC3 substrates have been previously reported as caspase-3 substrates and whether they are associated with apoptotic or non-apoptotic caspase activity. We consulted four sources of known caspase-3 substrates: MEROPS (Rawlings et al., 2010), CASBAH (Lüthi and Martin, 2007), DegraBase (Crawford et al., 2013), and Victor et al., 2018. We used a hypergeometric test to calculate the probability of obtaining the observed number of proteins that were caspase-3 substrates both in the above databases and in our ABC3 dataset, assuming each is randomly selected from a brainstem proteome of 5,653 proteins containing 288 caspase-3 substrates. Nearly one third (91) of our 288 ABC3 substrates were present in at least one of the four datasets (*p* = 2.45 × 10^−10^), suggesting that caspase-3 cleaves many of the same proteins in the developing auditory brainstem as in other conditions (Figure 3, Table 2, Supplementary Data 2). Of the roughly two-thirds (197) of the ABC3 substrates not found in any of the four databases, three were chicken-specific proteins, but the remaining 194 have homologs in other species and are thus novel caspase-3 substrates.

**Table 2:** ABC3 substrates overlap with known caspase-3 substrates.

Set	Set size	Expected ABC3 substrates in set	Observed ABC3 substrates in set	Fold enrichment	Enrichment *p*-value
**Apoptotic sets**
MEROPS	188	9.58	26	2.71	2.44E–06
CASBAH	397	20.2	38	1.88	1.22E–04
DegraBase Apoptotic	719	36.6	69	1.88	8.42E–08
**Total (Apoptotic)**	925	47.1	84	1.78	2.68E–08
**Non-apoptotic sets**
DegraBase Untreated	75	3.82	17	4.45	1.33E–07
Synaptosome	43	2.19	8	3.65	1.25E–03
**Total (Non-apoptotic)**	116	5.91	24	4.06	2.29E–09
**All sets**
**Total (All sets)**	953	48.55	91	1.87	2.45E–10

*Auditory brainstem caspase-3 substrates are overrepresented among sets of known caspase substrates, especially among non-apoptotic caspase substrates. For each set of known caspase substrates, set size indicates the number of proteins with orthologs detected in the chick brainstem proteome. Expected brainstem caspase-3 substrates were calculated by multiplying the set size by the fraction of total caspase-3 substrates in the proteome (i.e., 288/5,653 or 0.051). Fold enrichment is Observed/Expected brainstem caspase-3 substrates. The *p*-value for the number of observed brainstem caspase-3 substrates in each set was obtained with a hypergeometric test*.

Because the ABC3 dataset was obtained from embryonic brainstems not undergoing significant cell death (Rotschafer et al., 2016), we hypothesized that databases representing samples that are primarily apoptotic in nature (MEROPS, CASBAH, and the apoptotic DegraBase substrates) would contain fewer ABC3 substrates than sets more comparable to the present study insofar as caspase activity is localized to synapses (Victor et al., 2018) or is not associated with apoptosis (untreated DegraBase substrates). Our hypothesis was supported in that the proportion of ABC3 substrates in apoptosis-only datasets (67 / 837 = 8.0%) was less than that of non-apoptotic substrates, regardless of whether the proteins in non-apoptotic datasets were also found in apoptotic sets (24 / 116 = 20.7%; Figure 3, Fisher’s Exact Test, Odds ratio = 2.998, *p* < 0.0001). Additionally, the proportion of ABC3 substrates in non-apoptotic datasets did not depend on whether the non-apoptotic substrates were also found in apoptotic datasets (Odds ratio = 1.392, *p* = 0.5936). The same was not true for proteins in apoptotic datasets, which were more likely to be ABC3 substrates if they were also found in non-apoptotic datasets (Odds ratio = 2.752, *p* = 0.0014). These results show that the set of ABC3 substrates resembles known sets of caspase-3 substrates, especially those associated with non-apoptotic caspase activity.

### The Consensus Sequence of ABC3 Cleavage Sites Shows Both Similarities and Differences to Canonical Caspase-3 Consensus Sequences

**Figure 4:**
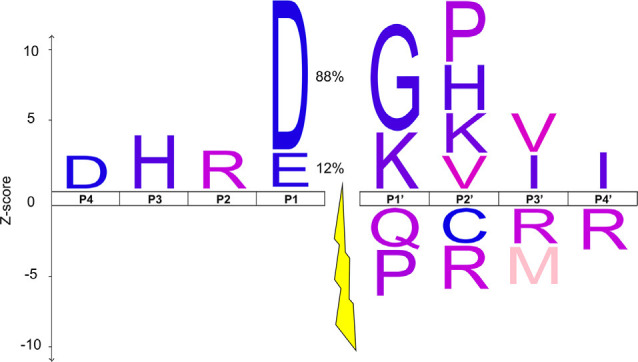
The IceLogo of the octapeptide sequence surrounding chick auditory brainstem D/E↓X cleavage events that were abolished by caspase-3 inhibition. The x-axis shows the 4 amino acids N-terminal (P1–4) and C-terminal (P1’-P4’) of the cleavage site, which is represented as a gap with a lightning bolt (Schechter and Berger, 1967). The y-axis depicts the Z-score by which the observed frequency of each amino acid in the experimental set differs from its randomly sampled mean frequency in the chicken proteome, signified by the height of each amino acid’s single-letter code. The *p*-value cutoff was set to 0.007, such that the IceLogo contains 1 or fewer false amino acid discoveries on average (Benjamini and Hochberg, 1995). The y-axis does not apply to the P1 amino acids since this position was used as part of the selection criteria for likely caspase-3 substrates. Instead, the percentage of sites with an aspartate (D) or glutamate (E) at the P1 position is displayed.

The high degree of enrichment for non-apoptotic targets of caspase-3 among ABC3 substrates may be attributable to differences in the amino acid sequence surrounding potential cleavage sites, which is instrumental in determining caspase substrate preference (Seaman et al., 2016). We therefore sought to determine the extent to which our ABC3 cleavage sites (Supplementary Data 1) resemble caspase-3 cleavage site consensus sequences reported in the literature. We used IceLogo (Maddelein et al., 2015) to develop consensus sequences of the P4-P4’ region (Schechter and Berger, 1967) for all 411 unique cleavage sites (Figure 4). The ABC3 IceLogo shared several features with the known caspase-3 consensus sequence (D-E-V/T-D↓G/S/A-V/A-P/S-A; Stennicke et al., 2000; Rawlings et al., 2010; Agard et al., 2012; Crawford et al., 2013). Importantly, we identified a positive enrichment for aspartate (D) at P4, glycine (G) at P1’, and valine (V) at P2’, supporting the role of caspase-3 cleavage in generating the ABC3 termini *in vivo*. We also noted several differences between the ABC3 IceLogo and the canonical consensus sequence for caspase-3 substrates at P2–3 and P1’-4’. Among these differences was the absence of the P3 and P2 preferences for E and V, respectively, notable because the P1–4 positions are the most important for caspase substrate selectivity (Talanian et al., 1997; Thornberry et al., 1997; Crawford et al., 2013; Julien and Wells, 2017). The H and R that are instead preferred at these positions in our ABC3 substrates do not, however, match the cleavage site profile for any other known aspartic protease (Rawlings et al., 2010). The other aspartic proteases detected in the auditory brainstem (caspase-6, -8 and -9) have P1–4 consensus sequences of VEHD, LETD, and LEHD, respectively, in which no position except for the P1 aspartate matches the ABC3 consensus sequence. Our observed ABC3 sequence of DHRD therefore most closely resembles the consensus sequence of caspase-3.

### Deviations of the ABC3 Cleavage Site Consensus Sequence From the Canonical Caspase-3 Cleavage Site Sequence Are Associated With Cytoskeletal Substrates

To identify a possible function for the differences between the ABC3 and canonical caspase-3 consensus sequences, we used the Database for Annotation, Visualization, and Integrated Discovery (DAVID) Bioinformatics Resource 6.8 to detect functional annotation term enrichment among the 55 ABC3 substrates with H at P3 or R at P2, compared to the full set of 288 ABC3 substrates (Huang et al., 2009a,b). We found that only the term “structural constituent of cytoskeleton” was enriched according to our significance criterion, a Benjamini-adjusted *p*-value of less than 0.05 (Supplementary Data 3). The enrichment of this term corresponded to a five-fold over-representation of cytoskeletal proteins among ABC3 substrates with cleavage sites similar to the DHRD consensus sequence, suggesting that the ABC3 consensus sequence is disproportionately found in cytoskeletal proteins. Thus, while some major features of canonical caspase-3 cleavage sites are preserved in our ABC3 substrates, we also note key differences in cleavage site sequences that may be instrumental in driving the enrichment of specific non-apoptotic substrates in the ABC3 set.

### Auditory Brainstem Caspase-3 Activity Cleaves Proteins Associated With Extracellular Vesicles

**Figure 5:**
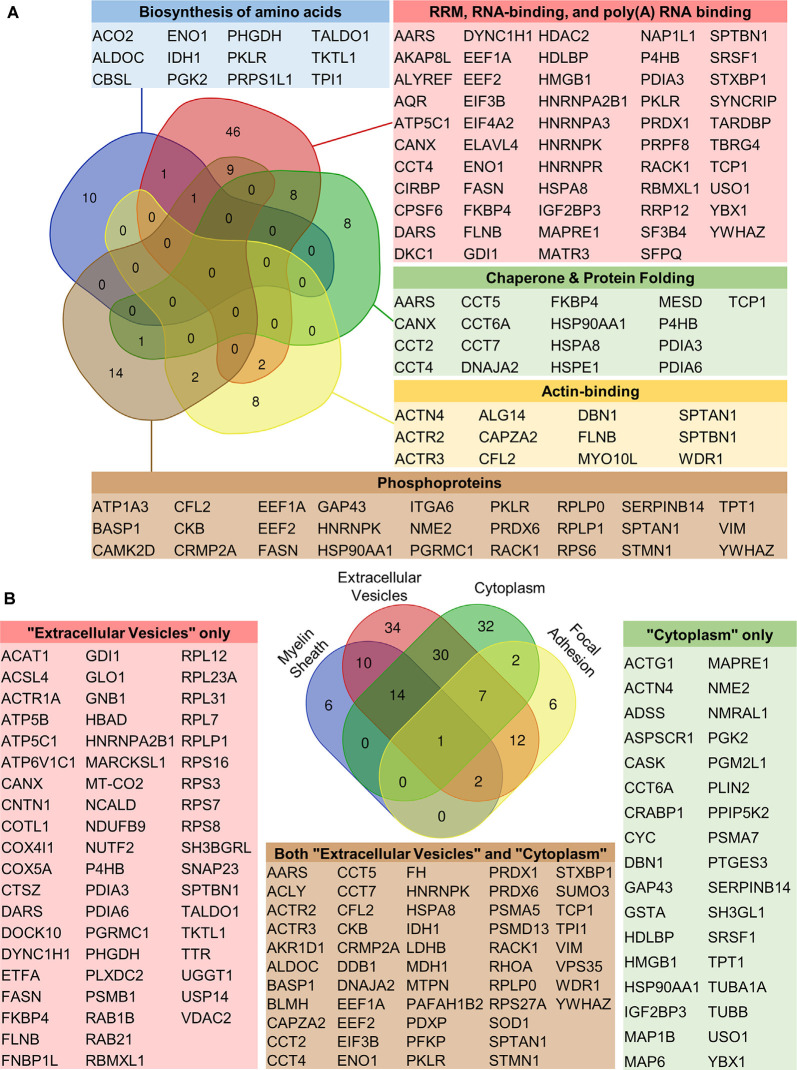
Functional annotation enrichment among auditory brainstem caspase-3 substrates. To determine the cellular and molecular pathways targeted by caspase-3 activity in the auditory brainstem, DAVID enrichment analysis was used to compare the frequency of functional annotation terms of ABC3 substrates to those of the auditory brainstem proteome. **(A)** ABC3 substrates were significantly enriched for six categories of functional annotation terms related to biological processes or molecular functions. Five of these categories and the gene symbols of their constituent ABC3 substrates are shown along with a Venn diagram depicting the number of ABC3 substrates in the categories and their overlaps. The sixth category, Ribosomal Proteins, contained 20 ABC3 substrates but is not shown due to its large overlap with the RNA binding category. Besides this exception, the ABC3 substrates in each category are generally unique to that category, suggesting that caspase-3 activity regulates several distinct processes during auditory brainstem development. Gene symbols of category members are shown in color-coded tables. Ribosomal proteins are not shown in the “RNA-binding” table but are included in the Venn diagram counts. **(B)** ABC3 substrates were significantly enriched for four terms related to cellular location. The Venn diagram shows the number of ABC3 substrates associated with each term. Two of these terms alone, Extracellular Vesicle and Cytoplasm/Cytosol, accounted for the majority of the proteins in the other two categories, suggesting that Extracellular Vesicles and Cytoplasm/Cytosol most parsimoniously describe the cellular location of the majority of ABC3 substrates. The overrepresentation of these categories among ABC3 substrates suggests that caspase-3 preferentially cleaves cytosolic proteins and extracellular vesicle (EV) proteins.

To determine the role of caspase-3 in the auditory brainstem, we next investigated pathways targeted by ABC3 activity. We again used DAVID to analyze functional annotation term enrichment among ABC3 substrates compared to the auditory brainstem proteome. Thirty-three terms were enriched among ABC3 substrates according to our significance criterion. These 33 terms were reduced to 14 terms after clustering of terms with similar protein content. Nine of these terms were related to biological processes and molecular functions of ABC3 substrates, representing six categories of pathways targeted by ABC3 activity: Biosynthesis of amino acids, RNA binding, ribosomal proteins, chaperones, and protein folding, actin-binding, and phosphoproteins (Figure 5A, Supplementary Data 3). Besides the inclusion of “ribosomal proteins” within “RNA binding,” these six categories generally overlapped little with each other, suggesting that ABC3 activity regulates several distinct processes.

Of the 14 post-clustering terms, the remaining five terms referred to the cellular location of the ABC3 substrates: Myelin sheath, extracellular vesicle, cytoplasm/cytosol, and focal adhesion (Figure 5B, Supplementary Data 3). Most ABC3 substrates in “myelin sheath” and “focal adhesion” were also found in “extracellular vesicle” and “cytoplasm/cytosol.” The reverse was not true, with many ABC3 substrates found only in “extracellular vesicle” and “cytoplasm/cytosol,” suggesting that these two terms best explain the cellular location of ABC3 substrates. The apparent enrichment of “focal adhesion” and “myelin sheath” proteins is likely a side effect of the similarity of these categories with “extracellular vesicle” and “cytoplasm/cytosol” combined with the substantial overrepresentation of proteins in the latter two terms. While we expected ABC3 substrates to be primarily cytosolic (since caspase-3 activity canonically occurs in the cytosol), we were surprised at the nearly 2-fold enrichment of extracellular vesicle (EV) proteins among our ABC3 substrates.

Extracellular vesicles are membrane-bound nanoparticles that facilitate intercellular signaling and transport of RNA, proteins, and other cargo between cells (Maas et al., 2017). Their molecular cargoes can induce changes in the recipient cell by altering gene expression with transported transcription factors, silencing translation with transported miRNAs, or other mechanisms (Yáñez-Mó et al., 2015; van Niel et al., 2018). Caspase-mediated cleavage of EV proteins has been previously described (Vardaki et al., 2016; Wang et al., 2016; Kim S. B. et al., 2017). However, EV-associated proteins are rarely exclusive to EVs (De Maio and Vazquez, 2013). Additionally, functional annotation terms like “Extracellular Vesicle” are assigned by DAVID and other annotation tools with varying levels of evidence, many of which are inferential but are given the same weight as direct observation (Balakrishnan et al., 2013). It was, therefore, possible that ABC3 substrates were enriched in EV proteins even in the absence of any caspase-3 activity associated with EVs or their contents.

**Figure 6:**
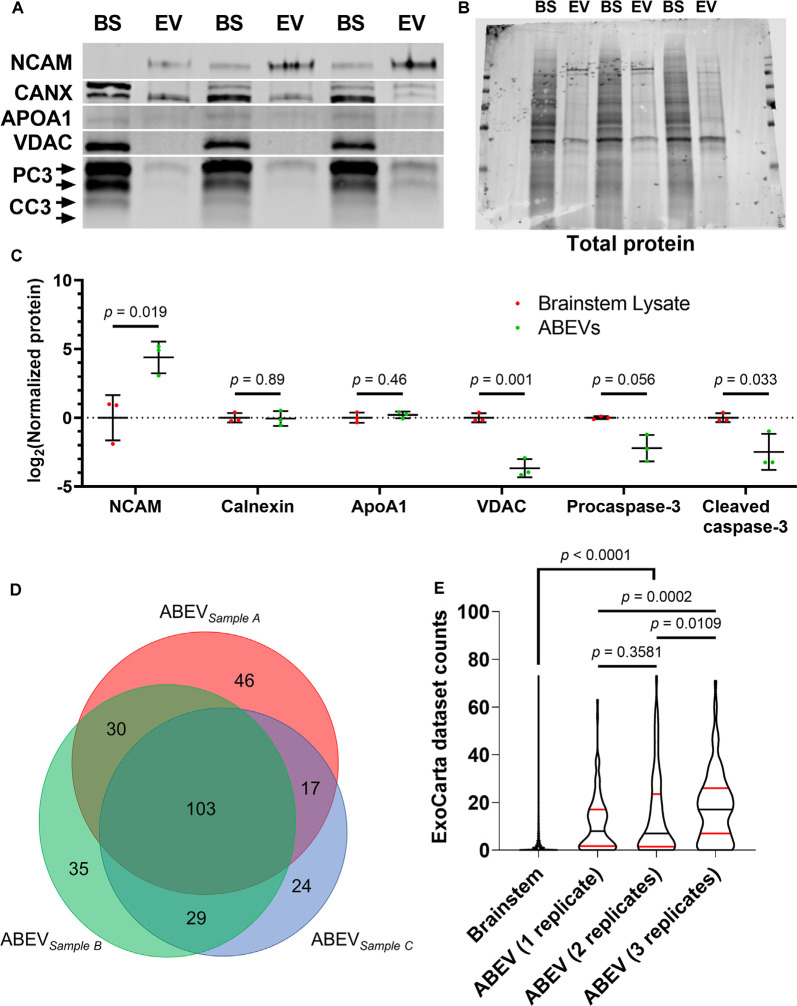
The purity of EV preparation. **(A)** Western blot comparing protein levels in three biological replicates of ABEVs (EV) and crude brainstem lysates (BS). Neural cell adhesion molecule (NCAM), a positive marker for EVs released by neural tissue, was enriched in ABEVs. In contrast, negative markers for EVs, namely calnexin (CANX), APOA1, and VDAC, showed either no difference in protein amount between brainstem lysates and ABEVs (CANX and APOA1) or showed a decreased expression in ABEVs (VDAC). Both procaspase-3 (PC3) and cleaved caspase-3 (CC3) were negatively enriched in ABEVs compared to brainstem lysates. **(B)** All proteins were normalized to total protein stain as a loading control. **(C)** Quantification of normalized protein levels from A. All *p*-values are derived from two-tailed Student’s *t*-tests except for procaspase-3, for which Welch’s heteroscedastic *t*-test was necessary. **(D)** Proportionally accurate Venn diagram of proteins identified in each ABEV replicate. The substantial triple-overlap reflects a high degree of conservation among the proteomes of the individual ABEV replicates. **(E)** Comparison of ExoCarta dataset counts of brainstem proteins and ABEV proteins found in one, two, or three replicates. The number of EV proteomic datasets containing each protein on the EV database ExoCarta (“ExoCarta dataset counts”) was used to estimate the frequency that proteins are found in EVs. ABEV proteins had higher ExoCarta dataset counts than brainstem proteins regardless of the number of replicates containing each ABEV protein. Additionally, ABEV proteins found in all three ABEV replicates had higher ExoCarta dataset counts than ABEV proteins found in only one or two replicates. These data suggest that the ABEV proteome resembles published EV proteomes. Plots depict smoothened probability density. Median (black horizontal line) and quartiles (red horizontal lines) are shown. *P*-values reflect Dunn’s multiple comparisons tests.

To explore the association of ABC3 substrates and EVs directly, we used size exclusion chromatography (SEC) columns (“Q26Voriginal” columns; IZON Science) to enrich for EVs from dissociated auditory brainstem tissue of E10 embryos. These columns are specifically designed to separate EVs from free protein, a capacity that has been independently shown to consistently yield high-purity samples of EVs in specific SEC fractions (Vogel et al., 2016; Stranska et al., 2018; Théry et al., 2018; Antounians et al., 2019; Brennan et al., 2020). E10 was chosen because ABC3 substrates were identified in the E10 brainstems. To quantitatively assess proteomic purity of our EV samples following the Minimal Information for Studies of Extracellular Vesicles (MISEV; Lötvall et al., 2014; Théry et al., 2018), we used Western blotting to compare levels of positive and negative EV markers in three biological replicates of auditory brainstem EV (ABEV) samples and crude brainstem lysates (Figures 6A,B). Neural Cell Adhesion Molecule, a positive marker for EVs released from neural tissue, was enriched more than 20-fold in ABEVs compared to crude brainstem lysates (Student’s* t*-test, *p* = 0.019). We also probed for several negative markers of EV purity, namely the mitochondrial outer membrane protein VDAC, the lipoprotein ApoA1, and the endoplasmic reticulum protein Calnexin (CANX). VDAC was present at lower levels in ABEVs than in brainstem lysates (*p* = 0.001). ApoA1 and CANX were present at the same levels in ABEVs and brainstem lysates (*p* = 0.46 and 0.89, respectively). It should be noted that EV-specific proteomes contain far fewer distinct proteins than tissue-specific proteomes, so any protein found in EVs is expected to comprise a larger portion of the EV proteome than of the tissue proteome (and thus to be enriched in EVs). Equivalent amounts of a protein in ABEVs and brainstem lysates (such as that observed for ApoA1 and CANX) therefore represent a relative depletion of that protein in ABEVs compared to the protein’s expected increased share of the ABEV proteome.

Next, we used tandem mass spectrometry to characterize the ABEV proteome. In the same three biological replicates of ABEVs, we identified a total of 284 characterized proteins (Figure 6C), 244 of which were previously detected in the proteomic screen used to identify ABC3 substrates (Supplementary Data 1). We detected several proteins that fulfill MISEV2018 requirements for EV proteomic characterization and purity, including heterotrimeric G proteins, annexins, cell adhesion molecules, cytoskeletal proteins, and a heat shock protein (Table 3). While we detected the negative EV marker APOB, it was observed at low Mascot scores in only two of three replicates, consistent with minor lipoprotein contamination often seen when purifying EVs by SEC (Stranska et al., 2018; Brennan et al., 2020). Taken together with our findings from immunoblotting, these results show that ABEVs are enriched for positive markers of EVs and largely devoid of negative markers of EVs, consistent with a relatively pure EV preparation with no major co-isolating contaminants.

**Table 3:** Identification of positive and negative EV markers in ABEV proteome.

MISEV category	Subtype	Proteins found (number of samples)
Positive EV markers 1- Transmembrane or GPI-anchored proteins associated with plasma membrane and/or endosomes	1a: non-tissue specific.	*Heterotrimeric G proteins*: GNAO1 (3), GNAQ (2), GNA11 (2) *Integrins*: ITGA6 (2) EMMPRIN / BSG (1)
	1b: cell/tissue specific.	Neural cell adhesion molecule / NCAM (3) Neuronal-glial cell adhesion molecule / NgCAM (3)
Positive EV markers 2- Cytosolic proteins recovered in EVs	2a: with lipid or membrane protein-binding ability.	*Annexins*: ANXA2 (3), ANXA5 (3), ANXA6 (3) Heat shock proteins: HSC70 / HSPA8 (3) Syntenin / SDCBP (2)
	2b: promiscuous incorporation in EVs (and possibly exomeres).	*Cytoskeleton*: actin (3), tubulin (3) *Enzymes*: L-lactate dehydrogenase (2), alpha-enolase (1), pyruvate kinase (1), transketolase (1)
Negative EV markers 3- Major components of non-EV co-isolated structures	3a: lipoproteins (produced by the liver, abundant in plasma, serum).	Apolipoprotein-B / APOB (2)
	3b: protein and protein/nucleic acid aggregates.	N/A

*Several protein markers of EVs, both cytosolic and membrane-bound, were detected by mass spectrometry of ABEV samples. MISEV2018 requires that proteomic studies of EVs demonstrate the presence of at least one protein each in categories 1 and 2, along with the absence, or depletion of proteins in category 3. We showed that all three ABEV replicates contain multiple proteins in categories 1 and 2. The only contaminant (category 3) protein detected by mass spectrometry, APOB, was detected at low Mascot scores in only two of three ABEV replicates, suggesting minor apolipoprotein contamination*.

To obtain further evidence on the extent to which our ABEV proteome resembles published EV proteomes, we next turned to ExoCarta, a database that compiles transcriptomic, proteomic, and lipidomic datasets from studies of extracellular vesicles (Mathivanan and Simpson, 2009; Mathivanan et al., 2012; Simpson et al., 2012; Keerthikumar et al., 2016). We used the number of EV mass spectrometry datasets represented in ExoCarta that contain each protein, designated “Dataset counts,” as an estimate of the relative frequency that EV proteomic studies have identified each protein (Supplementary Data 4). We compared the ExoCarta dataset counts of the brainstem proteome (*n* = 5653) to those of ABEV proteins found in 1 (*n* = 106), 2 (*n* = 77) or all 3 (*n* = 103) ABEV replicates (Figure 6D). A Kruskal–Wallis test found that the ExoCarta dataset counts significantly differed among these four groups (*p* < 0.0001). ABEV proteins were found in substantially more ExoCarta datasets than brainstem proteins (median = 0, 95% CI: 0–0), regardless of replicate count (Dunn’s multiple comparisons tests, *p* < 0.0001). Additionally, ABEV proteins found in one and two replicates had the same ExoCarta dataset count (*p* = 0.93), while ABEV proteins found in all three replicates had more than double the median dataset count (median = 17, 95% CI: 14–20) both of proteins found in two ABEV replicates (median = 7, 95% CI: 4–15; *p* = 0.0156) and of proteins found in one ABEV replicate (median = 8, 95% CI: 4–12; *p* = 0.0064). These results show that the ABEV proteome is highly similar to published EV proteomic datasets and with the strongest resemblance to published EV proteomes among proteins associated most strongly with the ABEV proteome (i.e., proteins found in all three ABEV replicates).

**Figure 7:**
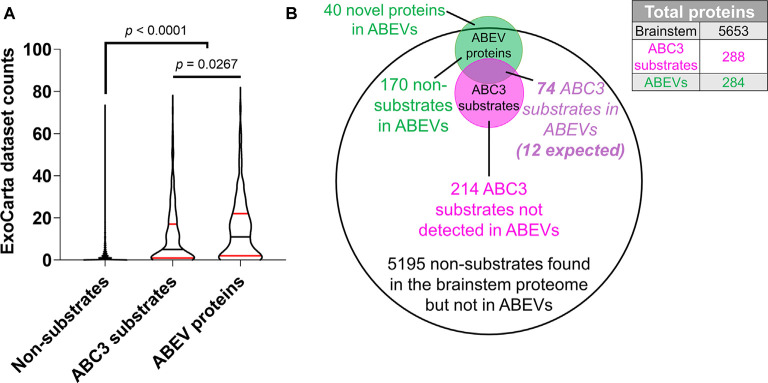
ABC3 substrates are enriched for EV proteins. **(A)** The number of EV proteomic datasets containing each protein on the EV database ExoCarta (“ExoCarta dataset counts”) was used to estimate the frequency that proteins are found in EVs. Both ABC3 substrates and ABEV proteins had higher ExoCarta dataset counts than non-substrates. Additionally, ABEV proteins had higher ExoCarta dataset counts than ABEV substrates, suggesting that ABC3 substrates are a mix of EV proteins and non-EV proteins. Plots depict smoothened probability density. Median (black horizontal line) and quartiles (red horizontal lines) are shown. *P*-values reflect Dunn’s multiple comparisons tests. **(B)** Proportionally accurate Venn diagram of brainstem proteins, ABC3 substrates, and ABEV proteins. The overlap of ABC3 substrates and ABEV proteins is expected to be about 12 proteins (288 × 244/5,653 = 12.4). However, 74 proteins were observed to be both ABC3 substrates and ABEV proteins, indicating that caspase-3 cleaves many proteins found in ABEVs.

We also sought to compare ExoCarta dataset counts of ABC3 substrates (*n* = 288) and non-substrates (*n* = 5,365) to those of ABEV proteins (*n* = 286) in order to determine the extent to which ABC3 substrates resemble ABEVs and published EV proteomic datasets (Figure 7A, Supplementary Data 4). A Kruskal-Wallis test showed that the three protein sets had different median ExoCarta dataset counts (*p* < 0.0001). ABC3 substrates (median = 5, 95% CI: 4–8) had higher ExoCarta dataset counts than non-substrates (median = 0, 95% CI: 0–0; Dunn’s multiple comparisons test, *p* < 0.0001), suggesting that ABC3 substrates resemble published EV proteomes more strongly than non-substrates. Additionally, ABEV proteins (median = 11, 95% CI: 8–14) had higher ExoCarta dataset counts than ABC3 substrates (*p* = 0.027), consistent with ABC3 substrates being a heterogeneous mix of EV and non-EV proteins.

We then directly compared the ABEV proteome to our ABC3 substrates to ascertain whether ABC3 substrates are enriched in ABEVs. Given a total brainstem proteome of 5,653 characterized proteins, a random overlap between the 244 previously observed EV proteins and the 288 ABC3 substrates would be expected to contain approximately 12 proteins (244 × 288/5,653 = 12.4). By contrast, we observed 74 proteins that were both ABEV proteins and ABC3 substrates, which represented a nearly six-fold enrichment (Figure 7B, hypergeometric test, *p* = 8.71 × 10^−40^). Thus, a large fraction (26%) of ABC3 substrates is detectable in the brainstem EV proteome, of which it composes a large proportion (30%). To test whether a protein’s strength of association with the ABEV proteome is related to its probability of being an ABC3 substrate, we also looked at the enrichment of ABC3 substrates among the individual sets of proteins found in one, two, or all three ABEV replicates. Each set showed heavy enrichment for ABC3 substrates (Table 4). However, the degree of enrichment differed among the three sets (Fisher-Freeman-Halton Exact Test, *p* = 0.032). This difference was primarily driven by greater enrichment of ABC3 substrates among proteins found in all three ABEV replicates (Fold enrichment = 7.85) compared to proteins found in only two ABEV replicates (Fold enrichment = 5.07; two-tailed Fisher’s Exact Test: *p* = 0.086) and to proteins found in only one ABEV replicate (Fold enrichment = 4.51; *p* = 0.017), not to the difference between proteins found in two and one replicates (*p* = 0.702). Thus, proteins found in all three ABEV replicates were more likely to be ABC3 substrates than proteins found in only one or two replicates, corroborating our model that caspase-3 cleaves EV proteins in the chick auditory brainstem.

**Table 4:** ABC3 substrates overlap with ABEV proteins.

Observed in—ABEV replicates	Proteins	Observed ABC3 substrates	Expected ABC3 substrates	Fold enrichment	Enrichment *p*-value
3	95	38	4.84	7.85	2.51E–25
2	62	16	3.16	5.07	4.44E–08
1	87	20	4.43	4.51	7.72E–09
**Any**	**244**	**74**	**12.43**	**5.95**	**8.71E–40**

*ABC3 substrates are overrepresented among ABEV proteins, with especially strong ABC3 substrate enrichment for among proteins found in all 3 ABEV replicates. Each set of ABEV replicates includes only proteins that were previously identified in the brainstem proteome, since these are the only proteins that could overlap with ABC3 substrates. Expected brainstem caspase-3 substrates were calculated by multiplying the ABEV set size by the fraction of total caspase-3 substrates in the proteome (i.e., 288/5653 or 0.051). Fold enrichment is Observed/Expected ABC3 substrates. A hypergeometric test was used to calculate the *p*-value for the number of observed brainstem caspase-3 substrates in each set of ABEV proteins*.

Despite this evidence that ABEV proteins are ABC3 substrates, we found that both procaspase-3 and active, cleaved caspase-3 were depleted in ABEVs compared to crude brainstem lysates (Figures 6A–C; Dunn’s multiple comparisons tests, *p* = 0.056 and *p* = 0.033, respectively), suggesting that proteolysis of ABEV proteins does not occur in ABEVs, but rather that caspase-3 cleaves ABEV proteins either before their loading into EVs or after EV uptake by recipient cells.

#### EV Characterization

**Figure 8:**
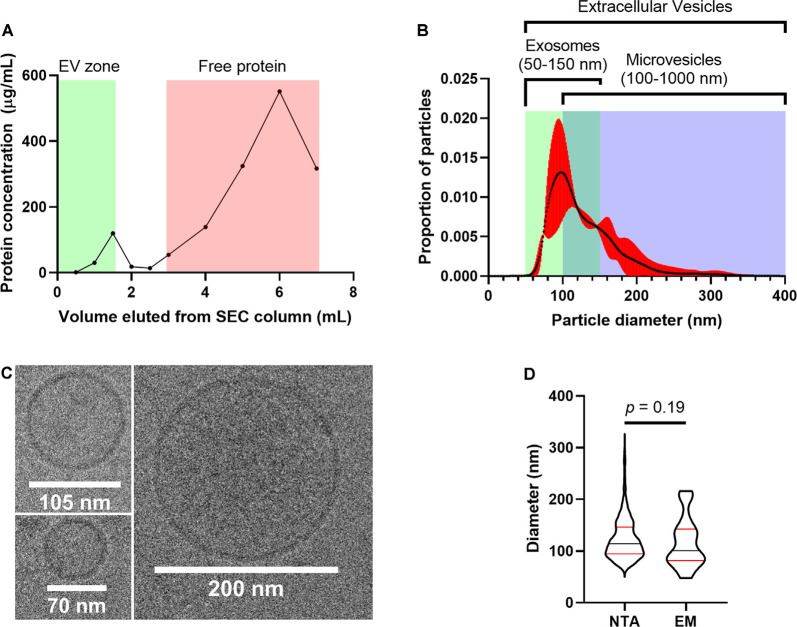
Embryonic chick auditory brainstems contain EVs. **(A)** The protein concentration of SEC fractions as a function of eluted volume. Fractions in the green “EV zone” are the SEC fractions that contain EVs, according to the Q26V column manufacturer (IZON Sciences). These fractions were pooled and used for ABEV analysis. **(B)** Size profile of ABEVs, generated using nanoparticle tracking analysis. The proportion of total particle number is shown as a function of particle diameter. The black curve is the mean of three biological replicates of ABEVs, and the red area is the 95% CI of the mean. The typical size ranges for two major types of EVs (exosomes and microvesicles) are depicted as well, showing that ABEVs likely contain a heterogeneous mix of EV subtypes. **(C)** Cryo-EM images of ABEVs. The lipid bilayer can be seen, as well as some electron-dense content. **(D)** Comparison of particle diameters derived from nanoparticle tracking analysis and cryo-EM. No difference was observed in the median particle diameter for the two methods (Mann-Whitney *U*-test, *p* = 0.191). Plots depict the smoothened probability density of distributions. Median (black horizontal line) and quartiles (red horizontal lines) are shown.

We used several additional approaches to characterize particles enriched from the auditory brainstem according to MISEV guidelines (Lötvall et al., 2014; Théry et al., 2018). ABEVs used for proteomic characterization were derived from SEC fractions within the “EV zone” based on the recommendation of the column manufacturer (IZON Science) and independent studies verifying this claim (Böing et al., 2014; Vogel et al., 2016; Stranska et al., 2018; Antounians et al., 2019; Brennan et al., 2020). Consistent with this expectation, a Lowry assay showed a peak in protein concentration that coincided with the EV zone fractions, as well as a peak that coincided with the “Free protein zone,” with relatively little protein in the fractions separating these two peaks (Figure 8A). The EV zone fractions were pooled and analyzed with nanoparticle tracking analysis (NTA), which measures the Brownian motion of particles in a sample to determine their size and concentration (Vestad et al., 2017) and cryogenic electron microscopy (cryo-EM) imaging (Figures 8B,C). NTA revealed that the pooled EV fractions contained particles with diameters ranging from 67 nm to 286 nm (1^st^ to 99^th^ percentile), with a peak in diameter at 99 nm (Figure 8B, Supplementary Data 5). Similarly, particles with a lipid bilayer, electron-dense content, and diameters ranging from 48 nm to 216 nm were observed by cryo-EM (Figure 8C). Particle diameters from NTA data corresponded to those from cryo-EM images, providing calibration for NTA analysis (Figure 8D; Mann-Whitney *U*-test, *p* = 0.191). Thus, particle sizes suggested that ABEVs consist of a heterogeneous mix of vesicles, including classically-defined exosomes (50–150 nm) and classically-defined microvesicles (100–1,000 nm; Gould and Raposo, 2013; Raposo and Stoorvogel, 2013; Colombo et al., 2014).

#### Caspase-3 Substrates Are Co-expressed With Cleaved Caspase-3 in Auditory Brainstem Structures During Development

**Figure 9:**
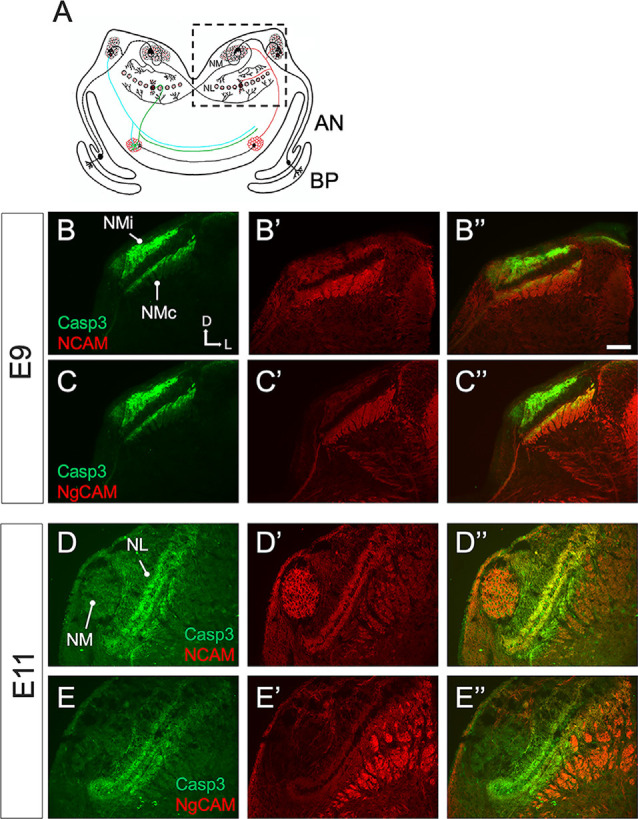
Caspase-3 and its substrates co-localize in the ITD circuit. **(A)** Schematic overview of a coronal section of the chick brainstem at the level of the auditory nuclei. Sound information flows from the basilar papilla (BP) through the auditory nerve (AN) to *Nucleus magnocellularis* (NM), which projects bilaterally to *Nucleus laminaris* (NL). The dashed-border rectangle shows the inset view in immunofluorescence images. **(B–B”)** Double immunofluorescence shows that cleaved caspase-3 and the ABC3 substrates Neural Cell Adhesion Molecule (NCAM) are both expressed in NM axons on E9. **(C–C”)** Double immunofluorescence also shows that cleaved caspase-3 and Neuronal-glial Cell Adhesion Molecule (NgCAM) are co-expressed in NM axons. **(D–D”,E–E”)** Cleaved caspase-3 and its substrates are expressed in NL dendrites on E11. Immunolabel of NCAM and Ng-CAM is seen both in NL dendrites and in non-auditory longitudinal axons seen in cross-section ventro-lateral of NL. NMi: NM ipsilateral axons. NMc: NM contralateral axons. Scale bar: 100 microns. Anatomical compass shows direction of Dorsal, Ventral, Lateral, and Medial.

Finally, we aimed to examine the expression patterns of ABC3 substrates during auditory brainstem development. Our screen for ABC3 substrates was conducted on chick brainstems in which caspase-3 activity had been inhibited on E9 and E10 when we observe strong cleaved caspase-3 expression exclusively in NM axons. We thus predicted that ABC3 substrates would also be present in NM axons at this time. We focused on NCAM and Ng-CAM (the chick homolog of mammalian L1CAM), because these two proteins were identified as both ABC3 substrates and ABEV proteins and because they have important roles in axon growth and guidance (Buchstaller et al., 1996; Westphal et al., 2010; Enriquez-Barreto et al., 2012). As expected, these ABC3 substrates were expressed in NM axons on E9 (Figures 9A,B–B”,C–C”). In contrast, the expression of NCAM and Ng-CAM in NM axons was absent at E11. Remarkably, it was instead observed in NL dendrites (Figures 9D–D”,E-”), a dramatic change in expression that parallels the previously demonstrated developmental shift in cleaved caspase-3 expression through the ascending chick auditory brainstem pathway (Rotschafer et al., 2016). This result suggests that caspase-3 may facilitate proper circuit formation at multiple auditory brainstem synapses through proteolysis of a similar set of substrates that may directly influence both axonal and dendritic morphology.

## Discussion

We previously showed that caspase-3 regulates normal axon targeting in the chick auditory brainstem (Rotschafer et al., 2016). Here, we used a proteomics approach to identify likely caspase-3 substrates that mediate this developmental role. We thus present, to our knowledge, the first non-apoptotic neurodevelopmental caspase degradome. Our DAVID and ExoCarta analyses revealed a heavy enrichment of extracellular vesicle proteins among ABC3 substrates. Many of our candidate substrates were proteins identified as caspase-3 substrates in other contexts, particularly in non-apoptotic proteomic analyses. We also showed that the number of ABEV replicates containing a protein was positively associated with both that protein’s frequency of appearance in EV proteomic datasets on ExoCarta and with the protein’s probability of being an ABC3 substrate, strongly suggesting that EV proteins and caspase-3 activity are related in the auditory brainstem. Finally, two ABC3 substrates present in ABEVs and involved in axon growth and guidance (NCAM and Ng-CAM) followed the same unusual developmental expression pattern as cleaved caspase-3, sequentially ascending the auditory pathway, corroborating the possibility that caspase-3 regulates an EV-mediated developmental process.

### Many ABC3 Substrates Are Involved in Proteostasis of Cytoskeletal Elements

Our findings suggest a novel neurodevelopmental mechanism by which caspase-3 activity in association with EVs is important for axon guidance and maturation of auditory nuclei. However, the function of caspase-3 activity in this context is still undefined. A common theme among the enriched functional categories of ABC3 substrates is that they represent several stages of the life cycle of cytoskeletal proteins, including amino acid biosynthesis, translation, protein folding, and filament polymerization and depolymerization. A key component of this model is the chaperonin-containing T (CCT) complex, an 8-subunit chaperone required for the folding of actin and tubulin monomers (Willison, 2018). All eight CCT subunits were ABC3 substrates, and we detected six of the eight subunits in ABEVs (Supplementary Data 1). The CCT complex thus represents a potential node of control of cytoskeletal protein production that can be rapidly regulated by proteolysis, putting several other enriched categories of ABC3 substrates into a proper perspective.

Disassembly of the cytoskeleton is a major cellular event during apoptosis. Actin was one of the first *in vivo* caspase-3 substrates identified (Mashima et al., 1997), and many caspase-3 substrates that regulate actin and microtubule dynamics have since been discovered (Lüthi and Martin, 2007; Crawford et al., 2013). Cytoskeletal degradation is known to be important for caspase-dependent axon guidance as well (Kellermeyer et al., 2018). For instance, degradation of spectrin by caspase-3 is required for NCAM-mediated neurite projection in cultured neurons (Westphal et al., 2010). In addition to cytoskeletal disassembly, caspases can stabilize the cytoskeleton by cleaving proteins responsible for cytoskeletal degradation and depolymerization, such as the proteasome and Actin-interacting protein 1 (Campbell and Holt, 2003; Li et al., 2007). The dual abilities of caspases to shape the assembly and disassembly of the cytoskeleton are especially useful in enabling the integration of attraction and repulsion (Gu et al., 2017; Kellermeyer et al., 2018), which lends greater sensitivity, complexity, and temporal precision to growth cones’ response to guidance cues (Buck and Zheng, 2002; Ming et al., 2002; Kaplan et al., 2014). Non-apoptotic caspase activity may therefore be a major way of achieving spatial precision in specialized synapses, such as those in the auditory brainstem.

### Selective Proteolysis of Caspase Substrates During Non-apoptotic Processes

Our ABC3 substrate cleavage site characterization (Figure 4) suggests that caspase activity might be restricted to specific substrates by the alteration of cleavage site preference. Caspase phosphorylation is known to differentially alter the cleavage rates of various substrates (Parrish et al., 2013; Zamaraev et al., 2017; Thomas et al., 2018), resulting in a consensus sequence different from that observed for unmodified caspase-3. Additionally, we observed a strong enrichment for “Phosphoproteins” among ABC3 substrates (Supplementary Data 3), so it is possible that different phosphorylation states among ABC3 substrates uniquely alter their susceptibility to caspase proteolysis without affecting the proteolysis of non-phosphorylated proteins, as has been observed in other contexts (Dix et al., 2012; Turowec et al., 2014; Kumar and Cieplak, 2018). Further research on the various post-translational modifications of caspases and their substrates will be necessary to determine if they result in changes to cleavage site preference.

### Functions of Caspase-3 in EVs

Caspase-3 cleavage of EV contents is known to influence intercellular communication by altering EV properties. For instance, caspase-3 must cleave the anti-apoptotic protein Bcl-xL in bone marrow stromal cell exosomes before they can be uptaken by myeloma and lymphoma cells, where the exosomes’ pro-apoptotic content paradoxically contributes to cell growth and proliferation (Vardaki et al., 2016). It is also possible that caspase-3 proteolysis regulates the properties of ABEVs by determining which proteins are loaded into EVs and therefore what properties the EVs possess concerning recipient cells (Sirois et al., 2011; Kim S. B. et al., 2017). For example, cleavage of translationally controlled tumor protein (TCTP) by caspase-3 is required for the sorting of TCTP into exosomes of apoptotic endothelial cells. These exosomes then exhibit a TCTP-dependent anti-apoptotic effect on neighboring cells, showing that caspase proteolysis of EV proteins has functional consequences even during apoptosis, when EVs are thought to function largely as a means to avoid death by removing active caspase-3 from cells (Trokovic et al., 2012; Böing et al., 2013).

The signaling roles of ABEVs may resemble those in other neural contexts (Sharma et al., 2013; Rajendran et al., 2014; Basso and Bonetto, 2016; Zappulli et al., 2016). Numerous studies have documented the ability of EVs to induce neural regeneration after injury (Ching et al., 2018; Bucan et al., 2019; Chen et al., 2019; Ma et al., 2019; Madison and Robinson, 2019; Xia et al., 2019). Recent work has shown that EVs likely function in every major aspect of neurodevelopment. Several neurodevelopmental deficits in a mouse model of Rett’s disease are rescued by treatment with EVs from non-Rett’s mice, which restore functions as diverse as neurogenesis, cell fate specification, synapse formation, and the establishment of normal firing patterns (Sharma et al., 2019). Additionally, EVs carrying the receptor tyrosine kinase EphB can direct contact-independent axon repulsion by canonical receptor-ligand (EphB-ephrinB) reverse signaling (Gong et al., 2016; Zhao et al., 2018). This finding is particularly relevant to the present study because our lab has shown that ephrins and Eph receptors are expressed throughout development by NM and NL projections, where they play major roles in midline crossing, axon guidance and segregation, and establishment of tonotopic gradients (Cramer et al., 2000, 2002, 2004; Person et al., 2004; Huffman and Cramer, 2007; Cramer and Gabriele, 2014). EphA5 is found in ABEVs (Supplementary Data 1), suggesting that ABEVs may have an important function in Eph-ephrin signaling in the chick auditory brainstem.

### Functional Transfer of RNA in EVs

Another major way that EVs influence neural development is by the transfer of genetic material between cells, including both mRNA and a wide variety of non-coding RNAs (ncRNAs) that can influence translation in recipient cells (Kim K. M. et al., 2017; Mateescu et al., 2017; Fowler, 2019; Luz and Cooks, 2019; Zhou and Chen, 2019). RNAs are selectively loaded into EVs by RNA-binding proteins (RBPs), which make up about 25% of EV protein content (Santangelo et al., 2016; Di Liegro et al., 2017; Hobor et al., 2018; Sork et al., 2018; Statello et al., 2018; Groot and Lee, 2020; Leidal et al., 2020). Our ABC3 substrates showed substantial enrichment for RBPs (Supplementary Data 3), including RBPs with known roles in loading RNAs into EVs, such as YBX1 (Shurtleff et al., 2016; Kossinova et al., 2017; Yanshina et al., 2018), SYNCRIP (Santangelo et al., 2016; Hobor et al., 2018), and HNRNP-A2B1 and -K (Villarroya-Beltri et al., 2013; Leidal et al., 2020). The most widely studied EV RNAs are miRNAs, which have diverse roles in regulating tumor growth, synaptic plasticity, recovery from stroke, and progression of neurological disease (van Balkom et al., 2013; Xin et al., 2013; Cheng et al., 2014; Lafourcade et al., 2016; Yang et al., 2016; Xiao et al., 2019). However, a recent transcriptomic study of EVs showed that miRNAs represent a minority of the ncRNAs in most EV populations, suggesting that we have barely scratched the surface of the functional transfer of RNA in EVs (Turchinovich et al., 2019). Little is known about the functional roles of other ncRNA subtypes in EVs (Dragomir et al., 2018; Driedonks and Nolte-’t Hoen, 2019; Kołat et al., 2019; Zhou and Chen, 2019). The importance of ncRNAs for many neurodevelopmental functions and the dysregulation of ncRNAs in neurodevelopmental conditions such as autism and schizophrenia suggests that EV-mediated functional transfer of ncRNAs may contribute to translational regulation in neurodevelopment (Sun and Shi, 2015; Gillet et al., 2016; Rajman and Schratt, 2017; Wang and Bao, 2017; Cho et al., 2019).

However, the mechanism by which EV-associated RNA can influence neurodevelopment remains unclear. A likely candidate is the localized regulation of axonal translation, which is necessary for growth cone navigation and has been implicated in a variety of neurological disorders (Holt and Schuman, 2013; Hornberg and Holt, 2013; Cioni et al., 2018; Koppers et al., 2019). Interestingly, the compartmentalization of translation in growth cones in response to guidance signals is also regulated by RBPs, which comprise 1% of all growth cone protein content (Estrada-Bernal et al., 2012; Holt and Schuman, 2013; Hornberg and Holt, 2013; Cioni et al., 2018). Caspase-3 proteolysis of RBPs, like other mechanisms that regulate RBPs (Hornberg and Holt, 2013; Cioni et al., 2018; Leidal and Debnath, 2020; O’Brien et al., 2020), may therefore serve two parallel functions in auditory brainstem development: Cleavage of RBPs that protect and compartmentalize RNA in growth cones, and cleavage of RBPs that load and transport RNA in EVs. RBPs such as the HNRNP family, six of which are ABC3 substrates, are known to be involved in both processes, so the two functions are not mutually exclusive (Liu et al., 2008; Glinka et al., 2010; Hentze et al., 2018; Lee et al., 2018; Statello et al., 2018; Thelen and Kye, 2020). Caspase-3 may thus influence translation in the growth cone and in neighboring cells simultaneously by cleaving a single RBP.

### EV-Mediated Regulation of the Apoptotic Pathway

Finally, another means by which EVs interact with caspases is the regulation of caspase activation in recipient cells. EVs, secreted both by apoptotic and non-apoptotic cells, are capable of suppressing caspase activity in their recipient cells, often with exosomal miRNAs that alter the expression of apoptotic regulatory proteins (Yang et al., 2013; Zhao et al., 2017; Li et al., 2019; Xiao et al., 2019; Wen et al., 2020). Instances of EVs activating caspases are less common, and the examples that exist occur through an unknown mechanism (Liu et al., 2018). In the simplest scenario, EV-associated caspase-3 may induce its own activation in recipient cells of EVs. During apoptosis, caspase activation occurs through trigger waves, in which a positive feedback loop is composed of caspase-3, caspase-9, and the X-linked inhibitor of apoptotic proteins (XIAP) allows for rapid dispersion of the apoptotic impulse throughout the whole cell (Cheng and Ferrell, 2018). Therefore, an additional benefit of caspase-dependent non-apoptotic processes is their potential for self-regeneration, which may be a mechanism by which caspase activity is transmitted between cells of the auditory brainstem (Rotschafer et al., 2016).

In summary, we have identified several hundred caspase-3 substrates that represent candidate mechanisms of auditory brainstem development, several of which involve communication between cells through EVs. Further work will be necessary to determine whether caspase-3-containing EVs from NM is responsible for the appearance of active caspase-3 in NL, and how EVs and their caspase-cleaved contents contribute to the circuit assembly.

## Data Availability Statement

The mass spectrometry proteomics data have been deposited to the ProteomeXchange Consortium (http://proteomecentral.proteomexchange.org) *via* the PRIDE partner repository (Vizcaino et al., 2013) with the dataset identifier PXD021728. The name of the repository and accession number can be found below: https://www.ebi.ac.uk/pride/archive/projects/PXD021728.

## Author Contributions

FW, YM, KS, MD, MB, and PG conducted experiments and collected data. FW, YM, MB, LC, PG, and KC analyzed and interpreted data and contributed to MS preparation. All authors contributed to the article and approved the submitted version.

## Conflict of Interest

The authors declare that the research was conducted in the absence of any commercial or financial relationships that could be construed as a potential conflict of interest.

## Footnotes

^1^http://bioinformatics.psb.ugent.be/webtools/Venn/
